# New insights into inhibition of human immunodeficiency virus type 1 replication through mutant tRNA^Lys3^

**DOI:** 10.1186/1742-4690-10-112

**Published:** 2013-10-24

**Authors:** Chengxiang Wu, Vivek R Nerurkar, Yuanan Lu

**Affiliations:** 1 Department of Public Health Sciences, John A. Burns School of Medicine, University of Hawaii at Manoa, 1960 East–west Road, Biomed. Bldg, D105, Honolulu, Hawaii 96822, USA; 2 Department of Microbiology, College of Natural Sciences, University of Hawaii at Manoa, 2538 McCarthy Mall, Snyder 207, Honolulu, HI 96822, USA; 3 Departments of Tropical Medicine, Medical Microbiology and Pharmacology, Asia-Pacific Institute of Tropical Medicine and Infectious Diseases. John A. Burns School of Medicine, University of Hawaii at Manoa, 651 Ilalo Street, BSB 325AA, Honolulu, HI 96813, USA

**Keywords:** Mutant tRNA^Lys3^, Reverse transcription, HIV-1 inhibition

## Abstract

**Background:**

Host cellular tRNA^Lys3^ is exclusively utilized by human immunodeficiency virus type 1 (HIV-1) as a primer for the replication step of reverse transcription (RTion). Consequently, the priming step of HIV-1 RT constitutes a potential target for anti-HIV-1 intervention. Previous studies indicated that a mutant tRNA^Lys3^ with 7-nucleotide substitutions in the 3′ terminus resulted in aberrant HIV-1 RTion from the trans-activation response region (TAR) and inhibition of HIV-1 replication. However, the mutant tRNA^Lys3^ also directed HIV-1 RTion from the normal primer-binding site (PBS) with potentially weakened anti-HIV-1 activity. To achieve improved targeting of HIV-1 RTion at sites not including the PBS, a series of mutant tRNA^Lys3^ with extended lengths of mutations containing up to 18 bases complementary to their targeting sites were constructed and characterized.

**Results:**

A positive correlation between the length of mutation in the 3′ PBS-binding region of tRNA^Lys3^ and the specificity of HIV-1 RTion initiation from the targeting site was demonstrated, as indicated by the potency of HIV-1 inhibition and results of priming assays. Moreover, two mutant tRNA^Lys3^s that targeted the IN-encoding region and *Env* gene, respectively, both showed a high anti-HIV-1 activity, suggesting that not only the TAR, but also distant sites downstream of the PBS could be effectively targeted by mutant tRNA^Lys3^. To increase the expression of mutant tRNA^Lys3^, multiple-copy expression cassettes were introduced into target cells with increased anti-HIV-1 potency.

**Conclusions:**

These results highlight the importance of the length of complementarity between the 3′ terminus of the mutant tRNA^Lys3^ and its target site, and the feasibility of targeting multiple sites within the HIV-1 genome through mutant tRNA^Lys3^. Intervention of the HIV-1 genome conversion through mutant tRNA^Lys3^ may constitute an effective approach for development of novel therapeutics against HIV-1 replication and HIV-1-associated diseases.

## Background

RTion, or the conversion of viral RNA (vRNA) into DNA, is a key step in the life cycle of HIV-1, and it may take place before budding as early as in virus producer cells [1,2]. The reaction is catalyzed by virion-associated reverse transcriptase (RT), and initiated with a cellular primer. Although various primer molecules can be used to initiate RTion *in vitro*, all retroviruses employ cellular tRNA [3-9]. DNA sequence analysis of the HIV-1 provirus reveals tRNA^Lys3^ to be the primer for HIV-1 RTion [10,11].

A prerequisite for the initiation of HIV-1 RTion is formation of a properly folded initiation complex of vRNA and tRNA^Lys3^. An 18-nucleotide residue at the 3′terminus of the tRNA^Lys3^ anneals complementarily to the PBS of vRNA, and primes template-dependent DNA synthesis [12]. Upon annealing, the primer is extended and a cDNA is synthesized and termed (-)strand strong-stop DNA or (-)ssDNA. The (-)ssDNA is released and anneals to the 3′ terminus of the vRNA, and primes further (-)strand DNA synthesis and generates a full-length (-)strand DNA that is used as a template for (+)strand DNA synthesis. Along with (-)strand DNA synthesis, RNaseH degrades the RNA template with the exception of two polypurine tracts (PPTs) that resist cleavage: one immediately upstream of the U3 region (3′-PPT) and the other at the center of the vRNA (cPPT). These PPTs are responsible for priming (+)strand DNA synthesis. The 3′-PPT-primed (+)strand DNA synthesis terminates at the first modified base in the tRNA^Lys3^ molecule and this product is termed (+)strand strong-stop DNA or (+)ssDNA [13,14], with the tRNA removed by RNaseH. A second strand-transfer takes place through annealing of the (+)ssDNA to the 3′ end of the full-length (-)strand DNA, followed by (+)strand DNA synthesis. Eventually, full-length double-stranded viral DNA is formed and integrates into the host cell genome through the viral integrase protein. For alpha and gamma-retroviruses and lentiviruses, these obligatory steps in genome conversion are chaperoned by a major virion protein of the inner core, the nucleocapsid protein encoded by Gag that serves as a key cofactor of the RT enzyme [15-21].

Different tRNAs are utilized by various retroviruses. Although many different tRNAs exist in an infected cell, each retrovirus is dedicated to its own tRNA [22-24]. For example, although a single point mutation in the HIV-1 PBS that results from the infrequent usage of a low abundant tRNA^Lys5^ variant has been observed [25,26], no spontaneous mutations or tRNA switches have been reported, except that primer specificity is less stringent for the murine leukemia virus [27-29]. Previous tRNA-switch study through forced selection of a HIV-1 variant that used a non-self tRNA primer- tRNA^Lys1,2^- resulted in severe replication defect, and reversion to the wild-type PBS-Lys3 sequence was the most frequent escape route [30].

Due to specific interactions between HIV-1 and tRNA^Lys3^, antiretroviral strategies targeting this unique property have been proposed and tested. TRNA^Lys3^ derivatives with mutations in their 3′-terminal sequence, were previously demonstrated to inhibit HIV-1 replication through induction of aberrant RTion products [31-33]. However, the described antiviral effect was minimal due to a limited alteration of the sequence. In this study, a series of mutant tRNA^Lys3^s were constructed with extended mutations in the 3′ terminus (up to 18 bp of complementarity to their targeting sites) with or without a combined A58U mutation. These mutants were shown to be encapsidated into progeny HIV-1 virions and reduced their infectivity. When the mutants were transduced into human lymphocyte-derived cells using an improved retroviral vector system [34], the transduced cells showed potent inhibition of HIV-1 replication, with the potency of anti-HIV-1 activity correlating with the complementarity between the mutated 3′ PBS-binding region of the mutant and its targeting site.

## Results

### Design and cloning of mutant tRNA^Lys3^ genes

**Figure 1:**
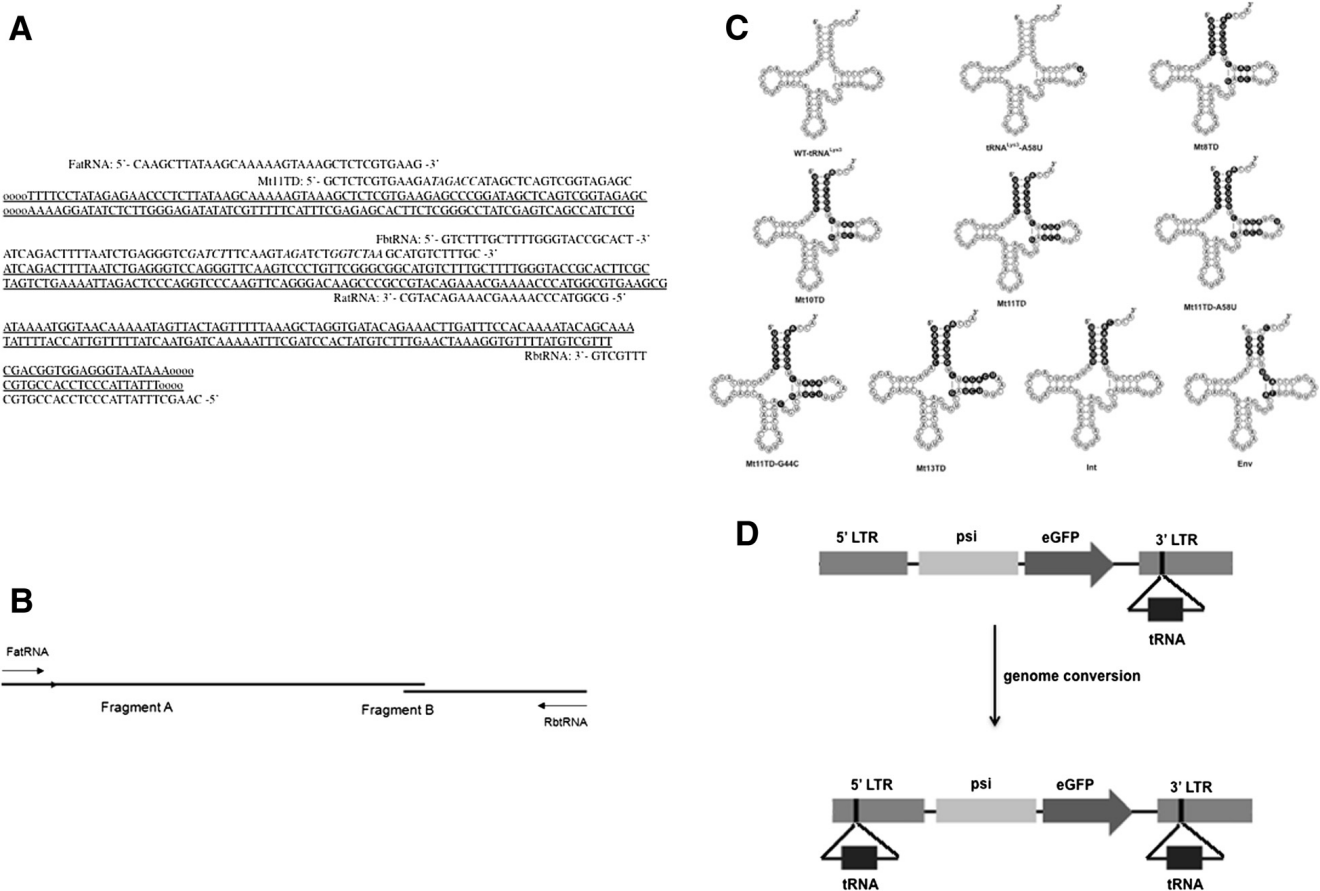
**Construction and retroviral vector-mediated delivery of mutant tRNA**^**Lys3**^**s. (A)** Sequence alignment of primer-template for the amplification of mutant tRNA^Lys3^ through PCR. The Mt11TD is used as an example and mutated bases are shown in bold italic**. (B)** Schematic illustration of the fusion-PCR used to amplify full-length mutant tRNA^Lys3^ genes. **(C)** Maps of the mutant tRNA^Lys3^s with mutated bases highlighted in darkened background. **(D)** Retroviral vector-mediated delivery of the mutant tRNA^Lys3^ genes.

To strengthen mutant tRNA^Lys3^-based anti-HIV-1 activity through extended mutation of the 3′ terminal sequence, and targeting other portions of the viral genome, mutant tRNA^Lys3^ genes with various length of mutation targeting either the TAR, IN-encoding region or *Env*, with up to 18 bases complementary to their target sites, were constructed through a fusion-PCR-based strategy (Figure 1A and B). Among these genes (Figure 1C), an 8-nucleotide mutation in the 3′ end of Mt8TD resulted in a 12-base pair (bp) complementarity to the TAR; a 10-nucleotide mutation in the 3′ end of Mt10TD conferred a 15-bp complementarity to the TAR. Similarly, an 11-nucleotide mutation in Mt11TD resulted in a total of 16-bp complementarity to the TAR, and a 13-nucleotide mutation in Mt13TD produced an 18-bp complementarity to the TAR. Besides these mutations, an extra A58U mutation in the Mt11TD-A58U was included to interfere with the termination of the (+)ssDNA product as previously reported [31,32]. In addition, an extra G44C mutation in Mt11TD-G44C was performed to examine if it was necessary to maintain the native secondary structure. Finally, two mutants, named Int and Env, were constructed with a 7-nucleotide mutation resulting in an 18-bp complementarity to the IN-encoding region and *Env* gene respectively. It is noteworthy that the CCA ends at the 3′ terminus of the wild-type and mutant tRNA^Lys3^s are added post-transcriptionally and are complementary to the binding sites. The number of mutated bases in each mutant and its complementarity to the targeting site are summarized in Table 1.

**Table 1:** **Length of mutation and complementarity, and targeting sites, of mutant tRNA**^**Lys3**^

**Name**	**Length of mutation**	**Length of complementarity**	**Targeting site**
Mt8TD	8	12	TAR
Mt10TD	10	15	TAR
Mt11TD	11	16	TAR
Mt11TD-G44C	11	16	TAR
Mt11TD-A58U	11	16	TAR
Mt13TD	13	18	TAR
Env	7	18	IN-coding region
Int	7	18	*Env*

To facilitate high efficiency and stable expression in human cells, these mutant tRNA^Lys3^ genes were cloned into an optimized double-copy retroviral vector (Figure 1D). Due to duplication of the 3′ U3 region during retroviral vector-mediated transduction of target cells, copy number of the mutant tRNA^Lys3^ gene in transduced cells is theoretically doubled as previously described [35,36].

### Retroviral vector-mediated transduction

**Figure 2:**
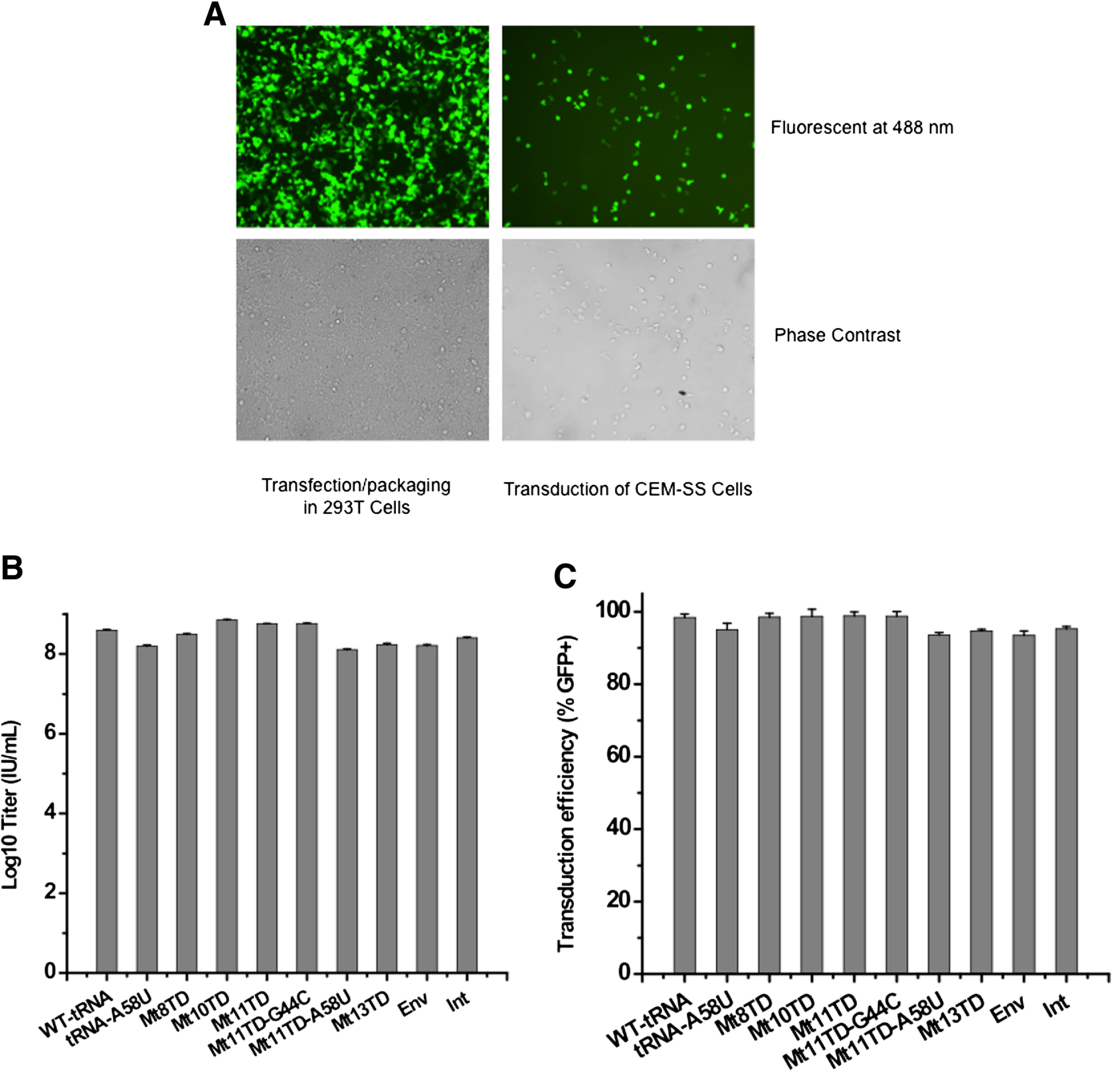
**Transduction of mutant tRNA^Lys3^ into CEM-SS cells. (A)** Vector production in 293T cells and transduction of CEM-SS cells based on eGFP expression. **(B)** Titers of the vector stocks concentrated through ultra-centrifugation. **(C)** High efficiency transduction of mutant tRNA^Lys3^ into CEM-SS cells.

A prerequisite for functional analysis of the mutant tRNA^Lys3^ is that they are expressed stably at a high level in human cells. This was accomplished through transduction of a human T lymphocyte-derived cell line, CEM-SS, with high-titer retroviral vector stocks. Since the retroviral vector carries an eGFP gene as a reporter [34], transfected 293T packaging cells and transduced CEM-SS cells were examined for eGFP expression (Figure 2A). To generate high-titer vector stocks for enhanced efficiency of gene transduction and expression, vector viruses harvested from transfected 293T cells were concentrated through a one-step ultracentrifugation method and vector titer exceeded 10^8^ IU/mL (Figure 2B). Comparative analysis showed that despite differences in titers of retroviral constructs containing different mutant tRNA^Lys3^ genes, no clear pattern of influence on vector production was observed, and the differences in titers were possibly due to variations among transfections. When the concentrated vector stocks were used to transduce CEM-SS cells at a multiplicity of infection (MOI) of 100, approximately 90-100% of the cells became GFP positive on day 3 post infection (pi) through a single transduction (Figure 2C). This allowed direct use of the transduced cells, without any selection or cell cloning, for functional analysis of the mutant tRNA^Lys3^ through HIV-1 challenging. Furthermore, transduction and expression of the mutant tRNA^Lys3^ were confirmed by PCR and RT-PCR (data not shown).

### Inhibition of HIV-1 replication

**Figure 3:**
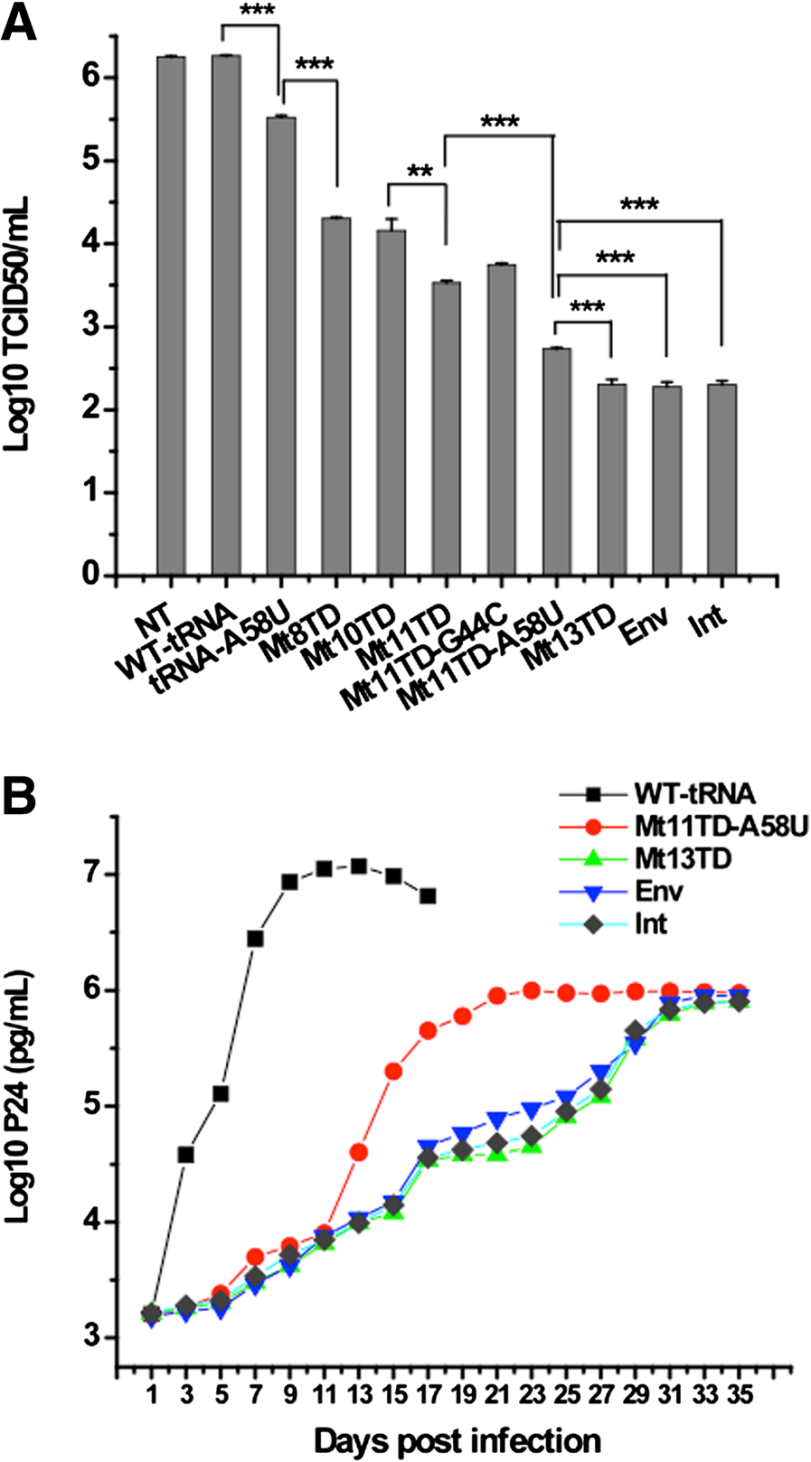
**HIV-1 challenging of CEM-SS cells expressing mutant tRNA^Lys3^s. (A)** TCID_50_ tests. **(B)** Challenging of cells that showed potent inhibition of HIV-1 in **(A)**. For cells transduced with the wild-type tRNA^Lys3^, sample collection and analysis did not go beyond day 17 due to massive cells death.

Anti-HIV-1 activities of the mutant tRNA^Lys3^ were firstly evaluated by examining the relative sensitivity of the transduced CEM-SS cells to HIV-1 infection and the capability of the cells to inhibit HIV-1 replication. This was done by infecting the mutant tRNA^Lys3^-expressing cells with a replication-competent HIV-1 stock, with relative capability of the cells to inhibit HIV-1 replication determined using the median tissue culture infectious dose (TCID_50_) assay. As shown in Figure 3A, the transduced cells expressing various mutants all showed significantly lower TCID_50_ titers of the HIV-1 stock compared with the non-transduced cells or cells transduced with the wild-type tRNA^Lys3^ (p < 0.001). Furthermore, cells expressing mutant tRNA^Lys3^ with increasing mutation in their 3′ PBS-binding regions generally showed significantly lower TCID_50_ titers (p < 0.001). Interestingly, cells transduced with the wild-type tRNA^Lys3^ did not significantly change their virus production (p > 0.05). Cells transduced with Mt11TD-A58U, Mt13TD, Int, and Env seemed the most refractory to HIV-1 replication, with significantly higher TCID_50_ reductions in cells expressing Mt13TD, Int, and Env than that in cells expressing Mt11TD-A58U (p < 0.001). Correspondingly, these four mutants had relatively more potent anti-HIV-1 activities than the others. To further analyze the anti-HIV-1 effects of these mutants, the transduced cells were infected with HIV-1 at MOI of 0.1, and cell-free supernatants from the infected cell cultures were tested for HIV-1 P24 production every two days for 35 days pi. Figure 3B shows that P24 accumulated rapidly from day 5 pi in control cells transduced with the wild-type tRNA^Lys3^ and reached a peak concentration of 1.2 x 10^7^ pg/mL on day 13 pi. In contrast, depending on the respective mutant expressed in the cells, the replication kinetics of HIV-1 was delayed by 3–10 days with significantly decreased production of P24 by 2–3 logs (p < 0.001).

### Multiple copy mutant tRNA^Lys3^ delivery

Using BLAST, twenty examples of a 234-bp sequence of the tRNA^Lys3^ gene were found in the human genome database (http://blast.ncbi.nlm.nih.gov/Blast.cgi?PAGE=MegaBlast&PROGRAM=blastn&BLAST_PROGRAMS=megaBlast&PAGE_TYPE=BlastSearch&SHOW_DEFAULTS=on&DBSEARCH=true&QUERY=&SUBJECTS=). Similarly, multiple copies of tRNA^Lys1^ and tRNA^Lys2^ genes were found. Therefore, we hypothesize if mutant tRNA^Lys3^ levels are increased in transduced cells, more mutant would be encapsidated into progeny HIV-1 virions upon infection, and the mutant tRNA-mediated anti-HIV-1 effect will be more effective because of enhanced competition against wild-type tRNA^Lys3^. To test this hypothesis, multiple copies of the Mt13TD gene (one of the mutants with potent anti-HIV-1 activity, Figures 3A and 3B) were subcloned into the retroviral vector and subsequently packaged and transduced into CEM-SS cells.

**Figure 4:**
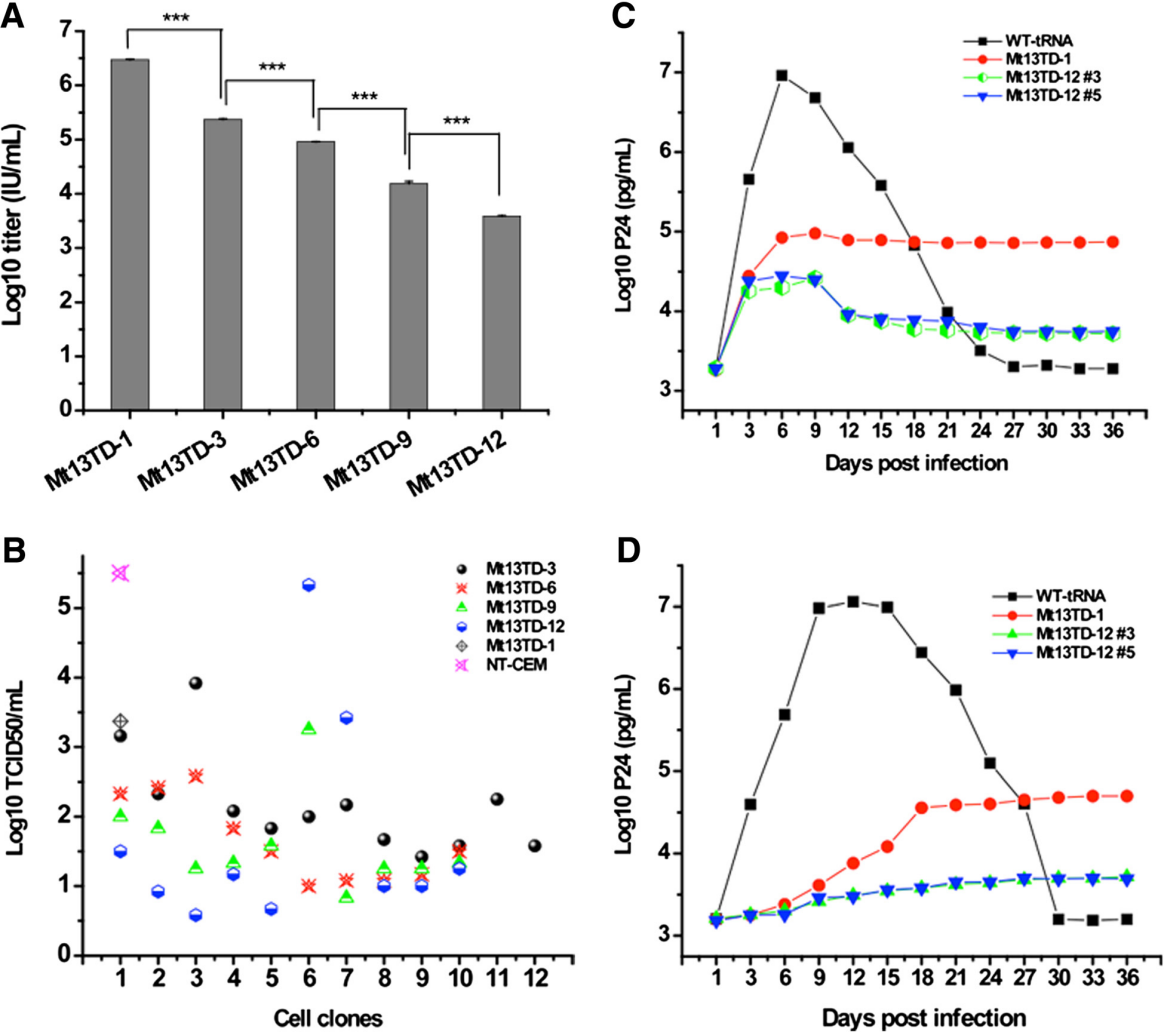
**Characterization of CEM-SS cells transduced with retroviral constructs carrying multiple copies of the Mt13TD gene. (A)** Comparison of titers of vector preparations. **(B)** TCID_50_ tests on cloned cells. Numbers of 3, 6, 9 and 12 correspond to copies of the gene in the retroviral constructs used to transduce the cells. NT, non-transduced. **(C)** Based on results in **(B)**, two clones of cells transduced with Mt13TD-12, namely No. 3 and No. 5 respectively, were challenged with HIV-1 at MOI of 1.0. Cells transduced with vectors containing one copy of either the wild-type tRNA^Lys3^ or the Mt13TD gene were challenged and sampled simultaneously as controls. **(D)** The same type of cells as in **(C)** were challenged at a lower MOI of 0.1 and sampled.

Although titers of retroviral vectors were not clearly influenced by insertion of single-copy of the mutant tRNA^Lys3^ genes, the retroviral constructs carrying multiple copies of the Mt13TD gene showed an apparent pattern of significant decrease in titers (p < 0.001) (Figure 4A). The vector titer from the triple copies of Mt13TD construct dropped from 3.03 ± 0.25 × 10^6^ IU/mL to 2.25 ± 0.35 × 10^5^ IU/mL compared with that of the construct carrying single-copy of Mt13TD– a 13-fold decrease, which was also seen where the titer derived from the construct with 12 copies of the Mt13TD gene dropped to 3.72 ± 0.12 × 10^3^ IU/mL. To overcome the challenge due to decreased titers from these vector constructs, clones of transduced cells were obtained through a limiting-dilution method and tested for viral inhibition with replication competent HIV-1.

In spite of marked variations among different clones, Figure 4B shows that cells transduced with the construct carrying 3 copies of Mt13TD showed lower TCID_50_ titers compared to cells transduced with single-copy of the gene. Consistently, cells transduced with the construct carrying 6 copies of the gene generally showed lower TCID_50_ titers than cells transduced with 3 copies. Furthermore, cells transduced with the construct carrying 12 copies showed the lowest TCID_50_ titers, with exception of two clones.

Based on the results from the TCID_50_ test, two of the clones transduced with the 12-copy construct were further evaluated by challenging with HIV-1 at MOIs of 0.1 and 1.0 respectively. As shown in Figures 4C and 4D, these two clones showed significant reduction of HIV-1 replication than cells transduced with a single copy of the same gene or the wild-type tRNA^Lys3^, especially the latter (p < 0.001). When challenged at MOI 1.0, peak production of P24 from cells transduced with the wild-type tRNA^Lys3^ occurred at day 6 pi with massive cell death, and P24 production decreased sharply following that time point. When these control cells were infected at MOI 0.1, the peak production of P24 occurred on day 12 pi, with the absolute concentration of the peak level 1.26 times higher than that of the cells infected at MOI 1.0. In contrast, when challenged at MOI 1.0, the cells transduced with the vector carrying single-copy of Mt13TD accumulated concentration of P24 2 logs lower with occurrence of the peak level delayed to day 9 pi. Following that time point, the P24 level remained relatively stable with a slight decrease. Similarly, when challenged at a lower MOI of 0.1, the P24 accumulated slower with concentrations greater than 2 logs lower compared with that of the cells transduced with the wild-type tRNA^Lys3^. In addition, the occurrence of peak concentration of P24 was delayed by 3 weeks, and the P24 levels remained relatively stable with only a slight increase following that time point.

In respect of challenging cells transduced by the construct carrying 12 copies of the Mt13TD gene at both MOIs, the data were drastically different. As shown in Figure 4D, in cells infected at MOI of 0.1, P24 accumulation occurred significantly slower compared to that in cells transduced with the wild-type tRNA^Lys3^ gene, with 3 logs lower concentration of P24 at its peak, and the P24 concentrations remained fairly stable. Moreover, these cells are much more potently inhibitory to HIV-1 replication compared to cells transduced with the vector containing single-copy of the Mt13TD gene. When challenged at MOI 1.0, the peak level of P24 was more than 2 logs lower compared to that of cells transduced with the wild-type tRNA^Lys3^ gene and 4 times lower than that of cells transduced with a single-copy Mt13TD (Figure 4C). Once the peak concentration of P24 occurred, P24 levels dropped to one log lower compared to that of cells transduced with a single-copy of Mt13TD.

### Potential adverse impact by mutant tRNA^Lys3^

**Figure 5:**
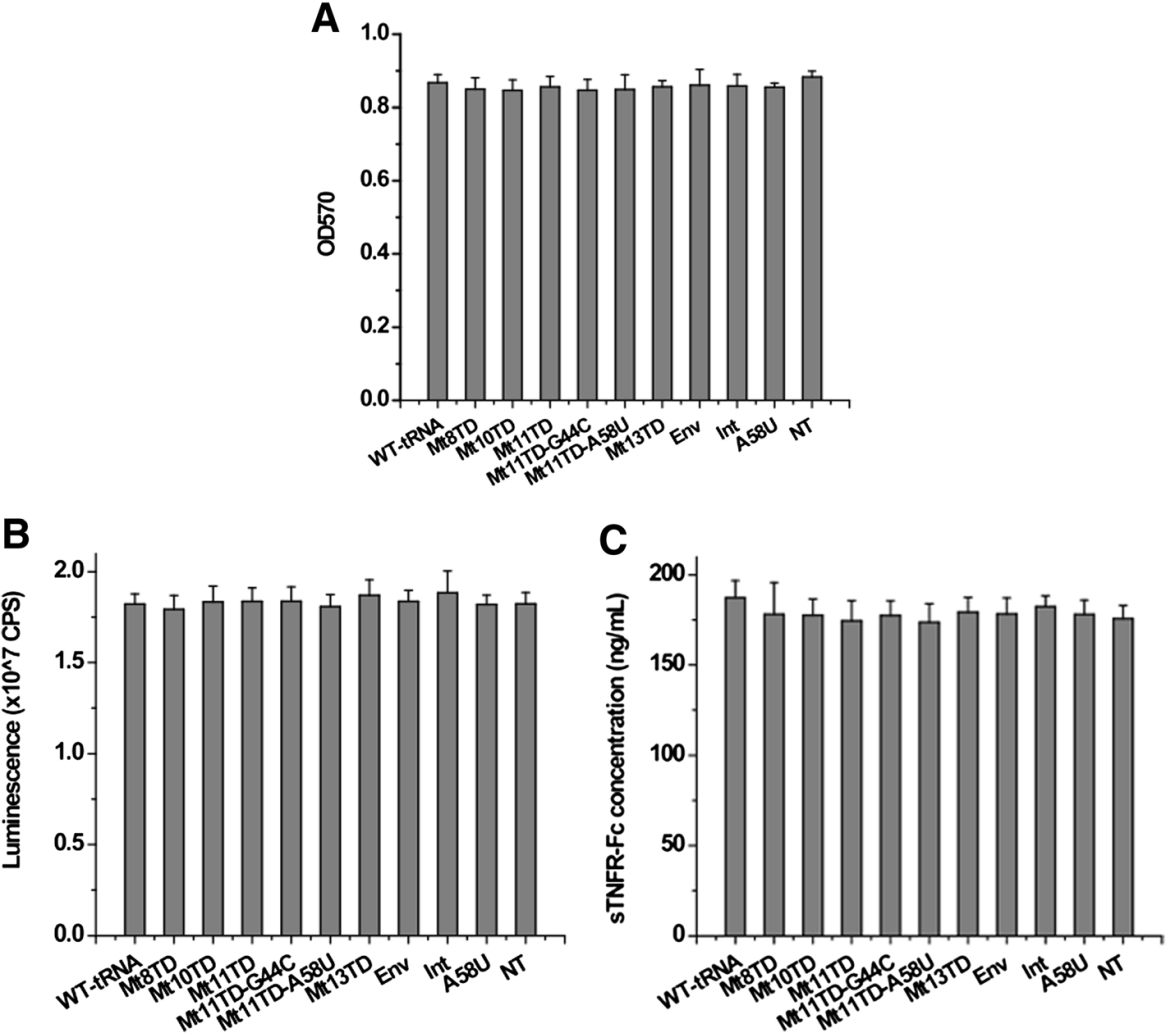
**Cytotoxicity assay. (A)** MTT tests on the growth and viability of CEM-SS cells transduced with mutant tRNA^Lys3^s; **(B)** Cells transduced as in **(A)** were infected with the same amount of lentiviral vector carrying the luciferase gene and tested for luminescence; **(C)** Cells transduced as in **(A)** were infected with the same amount of lentiviral vector carrying the hTNFR-Fc gene and tested for sTNFR-Fc in the supernatant.

To determine whether transduction and expression of mutant tRNA^Lys3^ resulted in any adverse impact on target cells, transduced cells were examined for their growth kinetics and morphology where no obvious alteration was found at different passage numbers (data not shown). Subsequent MTT assays also found no statistically significant difference between non-transduced cells and those transduced with mutant tRNA^Lys3^s (P > 0.05) (Figure 5A). To rule out the possibility that mutant tRNA^Lys3^ may interfere with the translation machinery in transduced cells, expressions of two reporter genes, luciferase [37] and hTNFR-Fc [38], were examined. Figure 5B and C indicate that expression of these reporter genes were not significantly influenced by the transduction and expression of mutant tRNA^Lys3^ (P > 0.05).

### Encapsidation and priming assay

**Figure 6:**
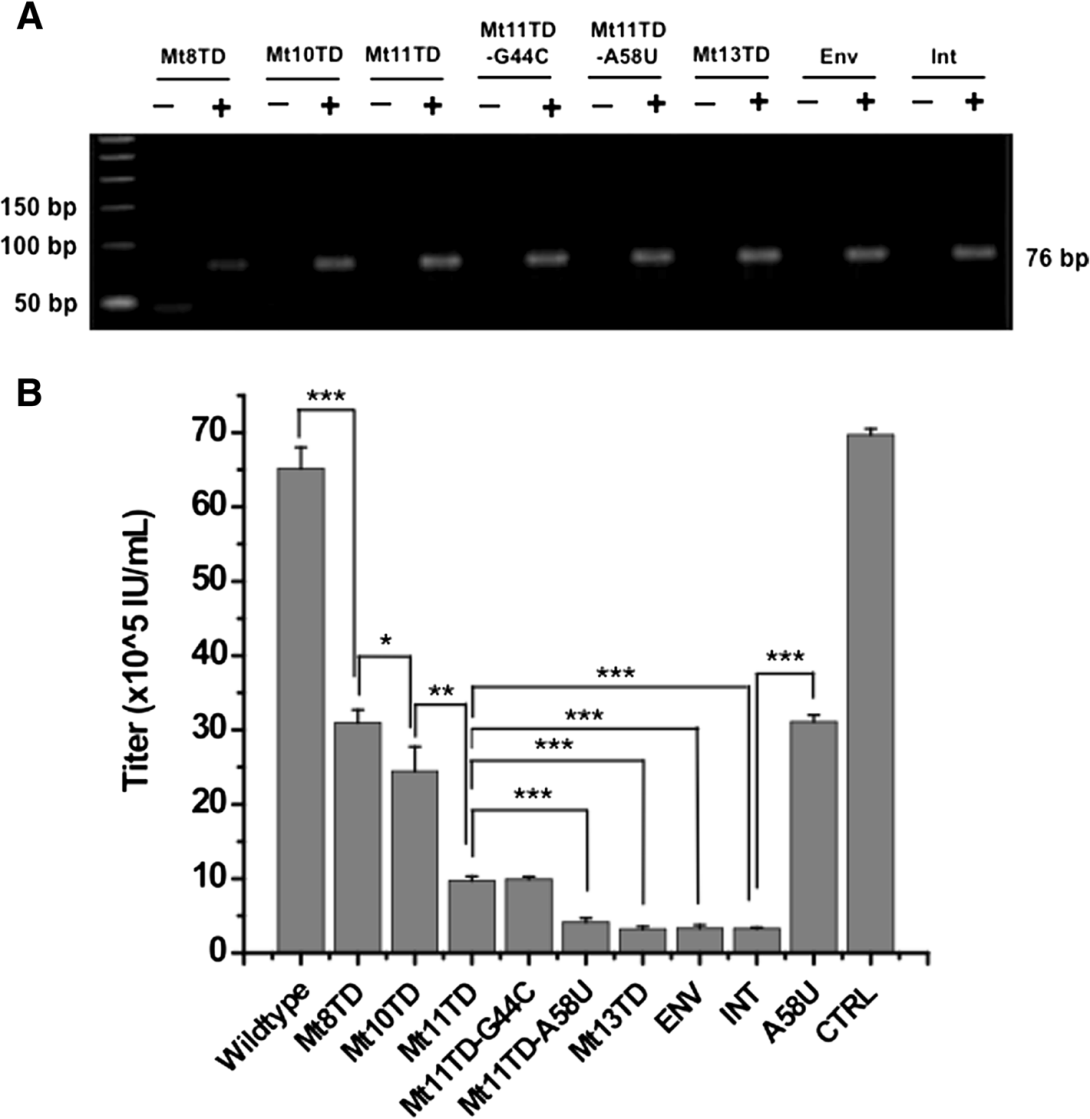
**Mutant tRNA^Lys3^ encapsidation assay. (A)** Detection of mutant tRNA^Lys3^ in HIV-1 virions that were generated through co-transfection of plasmids encoding a HIV-1-based vector system and a retroviral plasmid carrying the mutant tRNA^Lys3^ gene. +, with packaging plasmid; -, without packaging plasmid. **(B)** Titers of vectors generated through co-transfections as in **(A)**. Titers were determined on CEM-SS cells.

Analysis was performed at the molecular level to test the hypothesis that the improved anti-HIV-1 activities of the mutant tRNA^Lys3^s were conferred through improved priming or directing RTion of HIV-1 to their targeting sites rather than the normal PBS. Retroviral plasmid containing the mutant tRNA^Lys3^ gene was respectively co-transfected with a defective HIV-1-based vector system, with RT-PCR using RNAs extracted from the HIV-1 virions harvested from the co-transfections employed to examine encapsidation of the mutant tRNA^Lys3^s. As shown in Figure 6A, when the RT-PCR products were separated through 2.0% agarose gel electrophoresis, DNA bands of 76 bp corresponding to the size of the mutants were detected. When the HIV-1-based packaging plasmid was omitted from the co-transfections, the mutants were not detected under the same experimental conditions. This indicated that the mutant tRNA^Lys3^ genes were expressed from the retroviral constructs and encapsidated into the progeny HIV-1 particles.

In addition, their relative anti-HIV-1 activities were evaluated through a one-replication-cycle assay with replication-defective HIV-1-based vectors harvested from the co-transfections. This was accomplished through titration of the vectors in CEM-SS cells. Figure 6B shows that titers of the HIV-1-based vectors were significantly reduced when they were prepared through co-transfection with the mutant tRNA^Lys3^s (p < 0.001), with 15-20-fold reductions when the vector system was co-transfected with Mt11TD-A58U, Mt13TD, Env, and Int respectively. Moreover, a significant difference in the level of reduction was demonstrated, with a pattern consistent with the previous TCID_50_ assay (Figure 3A).

**Figure 7:**
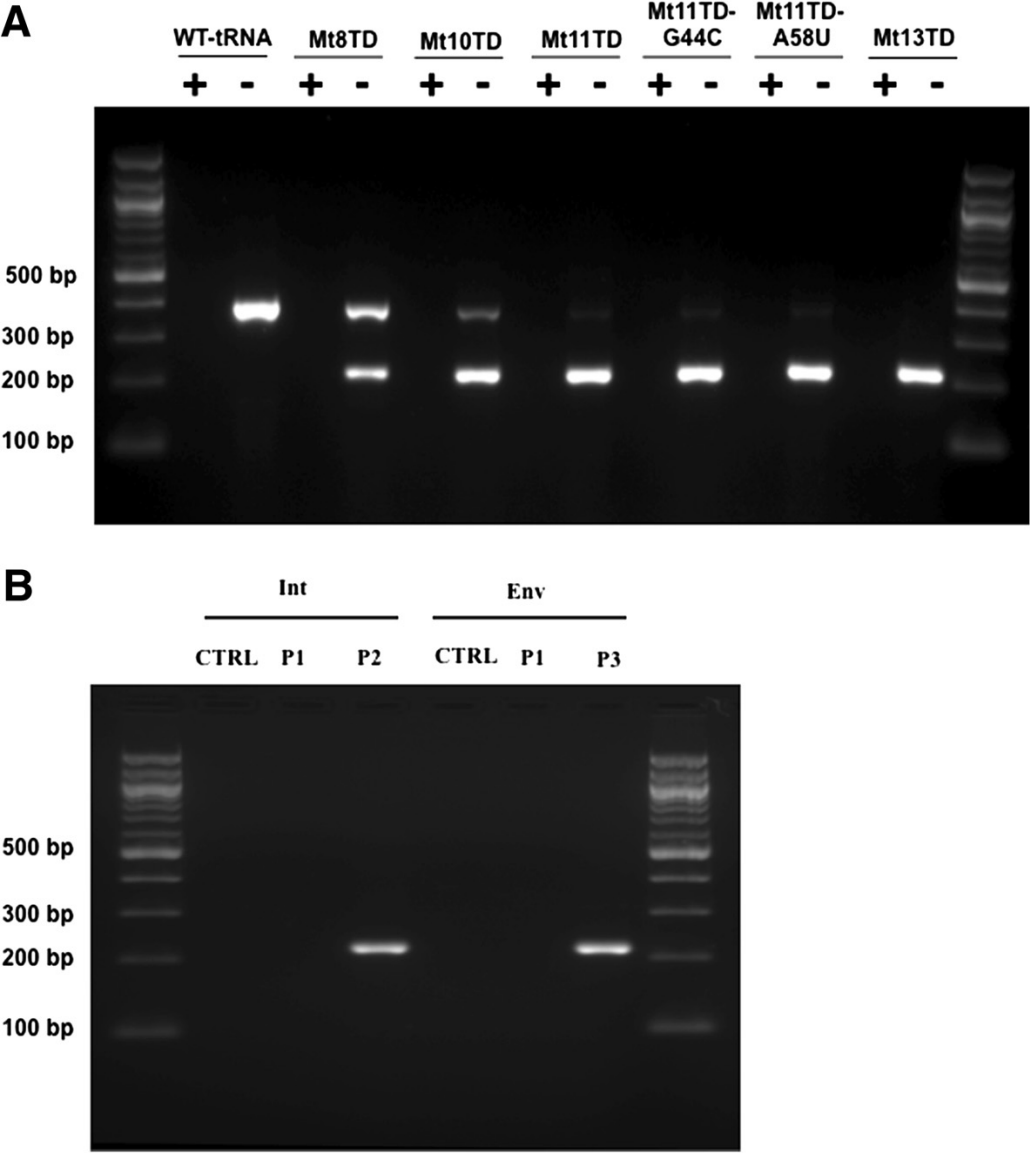
**Priming assay. (A)** Amplifications from the products of RTion primed through mutant tRNA^Lys3^s targeting the TAR. +, heat-inactiviation of HIV-1-based vectors before infection; –, no heat-inactiviation. **(B)** Amplifications from the products of HIV-1 RTion primed through mutant tRNA^Lys3^s targeting the IN-encoding region and *Env* gene respectively. CTRL, CEM-SS cells were infected with heat-inactivated HIV-1-based vector and RTion products were amplified using the combined primers for detection of both types of the RTion products.

To characterize the efficiency and specificity of mutant tRNA^Lys3^-primed HIV-1 RTion, the mutant tRNA^Lys3^-containing HIV-1 virions were used to infect CEM-SS cells, with DNAs extracted from the infected cells and subjected to PCR amplifications. Two primers, the forward primer specific to a site in the U3 region of HIV-1 3′ LTR of the RTion product following the first strand transfer event and the reverse primer specific to the mutated PBS-binding region of respective mutant tRNA^Lys3^, were used for the amplifications (more detail in Methods). For HIV-1 virions containing mutant targeting the TAR, PCR product amplified from RTion products primed from the TAR would be 226 bp in size. In case that the mutant tRNA^Lys3^ primes RTion from the normal PBS as previously reported [33], another PCR product with the expected size of 395 bp will be generated. Moreover, amplification efficiencies of the products would be approximately the same since the same primers were used and the PCR products are similar in size. Consequently, if both products were amplified, concentrations of the end products would reflect the relative starting amount of the templates. Figure 7A shows that Mt8TD made a fair amount of non-specific priming from the PBS, as indicated by the relative intensity of the 395-bp band to that of the 226-bp band. When the length of mutations was extended in other mutants, such as Mt10TD, non-specific priming was still detected but the relative intensity of the 395-bp band decreased notably. This tendency continued with Mt11TD. In case of Mt13TD that contained 18 bp complementarity to the TAR (Figure 1C and Table 1), the product that would reflected non-specific priming of RTion from the PBS was nearly non-detectable.

Similarly, priming of HIV-1 RTion by two of the mutants that target the IN-encoding region or *Env* gene was examined and characterized. Two PCR reactions were employed for each mutant (details specified in Methods). After separation of the PCR products, robust DNA bands amplified from the RTion products primed from their targeting sites were detected. In contrast, the PCR product that would reflect the RTion product primed non-specifically from the PBS was not detectable (Figure 7B).

### Quantification of transduction and expression of mutant tRNA^Lys3^

Due to significant variations in the anti-HIV-1 activities of different cell clones transduced with the multiple-copy Mt13TD constructs (Figure 4B), it was speculated that these variations might be caused by difference in the expression of Mt13TD among different clones. Real-time PCR and RT-PCR were employed to quantify the copy number of the Mt13TD gene and the relative expression level of Mt13TD versus that of the wild-type tRNA^Lys3^. Table 2 shows copy numbers of the Mt13TD gene in cells transduced with the multiple-copy constructs were largely determined by the vector constructs used, with exception for one cell clone transduced with vectors carrying either 3 or 9 copies of Mt13TD respectively, and two cell clones transduced with the construct carrying 12 copies of genes. However, levels of Mt13TD varied notably among cells transduced with the same vector construct. Among cell clones transduced with different multiple-copy constructs, relative expression of Mt13TD varied even more dramatically (Table 3). In particular, expression level of Mt13TD was less than that of the wild-type tRNA^Lys3^ in all cell clones regardless of the copy number of Mt13TD that was introduced into the cells. However, cell clones transduced with more copy numbers of Mt13TD generally tended to have higher expression levels, and this is consistent to their anti-HIV-1 activities as evaluated in previous tests (Figure 4B).

**Table 2:** Quantification of copy number of Mt13TD in cells transduced with the multiple-copy vector constructs

	**TD-1**	**TD-3**	**TD-6**	**TD-9**	**TD-12**
1	2.14 ± 0.17	6.08 ± 0.17	12.14 ± 0.23	18.05 ± 0.26	24.11 ± 0.19
2		5.98 ± 0.26	12.06 ± 0.28	18.03 ± 0.27	24.1 ± 0.27
3		4.07 ± 0.25	12.02 ± 0.13	18.04 ± 0.16	23.95 ± 0.23
4		6.07 ± 0.08	12.05 ± 0.24	18.07 ± 0.16	24.01 ± 0.13
5		6.04 ± 0.11	12.19 ± 0.17	17.99 ± 0.12	23.98 ± 0.17
6		6.07 ± 0.1	11.99 ± 0.26	13.08 ± 0.23	1.98 ± 0.17
7		6.02 ± 0.13	11.84 ± 0.47	18.06 ± 0.3	10.05 ± 0.09
8		6.01 ± 0.1	11.97 ± 0.1	18.0 ± 0.12	23.98 ± 0.17
9		5.98 ± 0.18	12.06 ± 0.25	18.03 ± 0.18	24.05 ± 0.08
10		6.06 ± 0.11	12.05 ± 0.11	18.1 ± 0.05	24.04 ± 0.15
11		6.09 ± 0.03			
12		5.95 ± 0.08			

Note: TD-1,3,6,9,12, retroviral vector carrying 1,3,6,9 and 12 copies of Mt13TD gene respectively; 1–12, cell clones derived from transduced cells with the multiple-copy retroviral constructs.

**Table 3:** Relative expression of Mt13TD versus wild-type tRNA^Lys3^ in cells transduced with multiple-copy vector constructs

	**TD-1**	**TD-3**	**TD-6**	**TD-9**	**TD-12**
1	6.91 ± 0.65	7.48 ± 0.79	20.74 ± 0.83	22.7 ± 0.41	27.01 ± 0.13
2		18.32 ± 0.63	17.4 ± 1.18	23.11 ± 0.34	57.87 ± 0.41
3		3.98 ± 0.26	15.48 ± 0.43	45.42 ± 0.33	82.35 ± 0.78
4		20.16 ± 0.81	22.66 ± 0.32	42.48 ± 0.38	48.68 ± 0.45
5		20.71 ± 0.81	26.15 ± 0.35	24.72 ± 0.18	77.26 ± 0.87
6		19.38 ± 1.13	48.74 ± 0.35	6.8 ± 0.39	0 ± 0
7		18.33 ± 1.22	43.43 ± 0.62	65.78 ± 0.76	4.11 ± 0.24
8		22.79 ± 0.41	41.1 ± 0.4	40.03 ± 0.45	54.37 ± 0.65
9		24.89 ± 0.23	38.54 ± 1.26	39.76 ± 0.64	55.74 ± 0.55
10		23.19 ± 0.35	23.14 ± 0.18	28.3 ± 1.03	35.18 ± 0.33
11		19.14 ± 0.83			
12		22.9 ± 0.15			

Note: the expression level of wild-type tRNA^Lys3^ is arbitrarily set as 100%, and the relative percentages of expression of Mt13TD are shown.

## Discussion

### Rationale for designing the mutant tRNA^Lys3^

tRNAs are an essential part of the protein translation mechanism in cells and are recognized by many intracellular proteins including the 5′ and 3′ tRNA processing enzymes [39] and tRNA aminoacyl transferases [40,41]. Retroviruses selectively encapsidate tRNAs as primers, resulting in an increased concentration of certain tRNAs inside the virions compared with the cytoplasm of the infected cells [42-45]. For the selective incorporation of tRNA^Lys^ into HIV-1, both the vRNA and interactions between the tRNA^Lys^ and vRNA are dispensable since viral particles lacking an RNA genome are still able to incorporate the wild-type set of tRNA^Lys^[45]. However, selective packaging of tRNA^Lys^ is affected in virions lacking a functional RT domain [45-47], and the centrally located thumb subdomain of RT is indispensable [48].

In HIV-1 virions, all tRNA^Lys^ isoacceptors are enriched. The ratio of tRNA^Lys3^ versus tRNA^Lys1,2^ are the same in cells and virions, with approximately 8 and 12 molecules per particle respectively [49]. The tRNA^Lys^ molecules are encapsidated during particle assembly via interactions between the Gag-Pol precursor and a protein complex composed of the cellular lysyl-tRNA synthetase (LysRS) and the Gag protein [45,48,50-52]. Changing the intracellular levels of LysRS, by either overexpression or siRNA-mediated silencing, results in a concomitantly altered level of tRNA^Lys^ in virus particles. This suggests that LysRS may be the limiting factor for tRNA^Lys^ packaging [53-55]. The presence of other tRNA synthetases in HIV-1 virions has also been analyzed [51,56], with only LysRS detected among eight synthetases screened. Approximately 20–25 LysRS and 20 tRNA^Lys^ molecules are present per virus particle, indicating an approximately equimolar stochiometry [51]. These previous studies provided the supporting foundation for this study of anti-HIV-1 replication by designing and constitutive expression of the mutant tRNA^Lys3^.

In designing the mutant tRNA^Lys3^, we extended the length of mutations in the 3′ terminal PBS-binding region to enhance their binding specificity and efficiency of directing the RTion of HIV-1 to new targeting sites. In addition, corresponding mutations to maintain the natural secondary structure of tRNA^Lys3^ were made (Figure 1C) with the natural promoter and terminator sequences needed for transcription and post-transcriptional processing maintained. Integrity of the anti-codon domain that is important for interactions between the tRNA^Lys3^ and LysRS was also kept intact. To facilitate efficient transcription and processing of the mutant tRNA^Lys3^ genes, the 5′and 3′ flanking sequences that were derived from three of the most efficiently expressed cellular tRNA^Lys3^ loci [57] were included. Consequently, except for the mutations mentioned, the remaining parts of the tRNA^Lys3^ were not altered (Figure 1C). Because both vRNA and interactions between tRNA and vRNA are dispensable in the tRNA^Lys3^ encapsidation process [45], encapsidation of the mutant tRNA^Lys3^ is unlikely to be influenced, as confirmed by RT-PCR tests (Figure 5A).

### Improved inhibition of HIV-1 replication

We report that mutant tRNA^Lys3^ with extended mutations in the PBS-binding region were effectively expressed and encapsidated into progeny HIV-1 virions and they redirected the RTion of HIV-1 to targeting sites with improved specificity and efficiency, which concomitantly heightened the inhibition of HIV-1 replication. Furthermore, besides the TAR, sites downstream of the PBS such as the IN-encoding region and *Env* gene could be effectively targeted. Moreover, enhanced anti-HIV-1 activity was observed when these mutations were combined with a previously reported A58U mutation [32]. This indicates the mutations in the 3′ PBS-binding region conferred anti-HIV-1 activities in a different mechanism from that of the A58U mutation, which could give rise to an additive anti-HIV-1 effect.

### Transduction and expression of mutant tRNA^Lys3^

For efficient delivery and expression of the mutant tRNA^Lys3^ in human cells, a retroviral vector system was optimized [34] and employed. The retroviral vector was used because it does not process inherent capability of inhibiting HIV-1 replication as previously demonstrated [32,33]. This offers an advantage for the analysis of anti-HIV-1 activities compared to a HIV-1-based vector that has been previously shown to process potent inherent inhibition of HIV-1 replication [58]. Furthermore, titers of the retroviral vectors with a single-copy of the mutant tRNA^Lys3^s were not significantly affected, indicating the mutants did not interfere with infectivity of the retroviral vector. Although titers of vector preparations were significantly reduced when multiple copies of Mt13TD were introduced, such reduction might not be directly related the mutant tRNA^Lys3^. Rather, a more plausible explanation is the insertion of a large DNA fragment in the LTR that might hinder vRNA processing and transduction of target cells as previously reported with lentiviral vectors [59]. Rather than being used as a delivery tool, an HIV-1-based vector system was employed for the one-replication-cycle infection assay and provided supplemental evidence to those obtained through HIV-1 challenging tests. When the mutant tRNA^Lys3^ constructs were co-transfected with the HIV-1-based vector system, vector titers were significantly reduced by more than one log for four of the mutants tested (Figure 6B). These results indicates a marked hindrance of the RTion process within the HIV-1 virions, which is consistent with potencies of the anti-HIV-1 activities of the mutants as determined by TCID_50_ and HIV-1 challenge tests (Figures 3A and 3B).

### Transduction of multiple copies of mutant tRNA^Lys3^

Naturally, all tRNA^Lys^ isoacceptors are enriched in the HIV-1 virions with the same the ratio of tRNA^Lys3^ versus tRNA^Lys1,2^ between cells and virions [49]. In addition, there are multiple copies of these genes in the human genome. To improve the encapsidation of mutant tRNA^Lys3^ through increasing its concentration in transduced cells, we hypothesized that delivery of multiple copies of the gene may further boost its anti-HIV-1 effects. As anticipated, transduction of multiple copies of the Mt13TD gene into CEM-SS cells resulted in decreased HIV-1 replication (Figures 4B and 4C). Furthermore, we examined the copy numbers of Mt13TD in these clones and the expression level of Mt13TD as compared to that of its wild-type counterpart. We demonstrated the potency of inhibition of HIV-1 replication in the cell clones was more closely determined by the relative level of expression of Mt13TD, rather than by the copy number of this gene that was introduced into the cells (Figure 4B and C and Tables 2 and 3). As for the variations in expression of the gene, it could be attributed to the position effect of the integration site. For the four clones with less copies of Mt13TD as expected, we hypothesize that some irregular event such as recombination was the cause, since retroviral vectors are unstable with repeat sequences [60]. Nevertheless, none of these is directly associated with the mutant tRNA^Lys3^ but are more likely to be associated with the delivery system. Future optimizations on the vector system or change of the delivery vector may possibly resolve these issues.

### Implications of targeting multiple sites

Due to high mutation rates of HIV-1, drug resistance mutations constitute a major concern that confronts current antiretroviral strategies. When mutant tRNA^Lys3^ targeting a single site of the HIV-1 genome is expressed, potential viral resistance is possible through mutations in the PBS to acquire complementarity to the mutant tRNA^Lys3^ or in the site being targeted to reduce its complementarity to the mutant tRNA^Lys3^. Therefore, the mutant tRNA^Lys3^-mediated anti-HIV-1 strategy would be more effective if multiple sites within the HIV-1 genome could be targeted. To test this concept, two tRNA^Lys3^ mutants targeting the IN-encoding region and *Env* gene, respectively, were tested. These targeting sites were selected because only a 7-base substitution in the 3′ PBS-binding region of the tRNA^Lys3^ allowed an 18-bp complementarity to the sites respectively (Figure 1A). RTion priming tests indicated that these mutants were as effective as others targeting the TAR (Figure 7B) with high specificity and efficiency, showing no detectable priming activity from the PBS. Both TCID_50_ assay and HIV-1 challenging tests indicated these mutants could lead to inhibition of HIV-1 infection to similar potencies as Mt13TD (Figure 3A and 3B). These findings clearly suggest that the mutant tRNA^Lys3^-mediated inhibition of HIV-1 replication is not limited to targeting sites upstream the PBS and other portions of the vRNA could be effectively targeted. Moreover, this makes it feasible for simultaneous delivery of multiple mutants that target various portions of the vRNA, which would provide a strong genetic barrier for spontaneous evolution of resistant HIV-1 genome.

### Proposed anti-HIV-1 mechanisms through mutant tRNA^Lys3^

**Figure 8:**
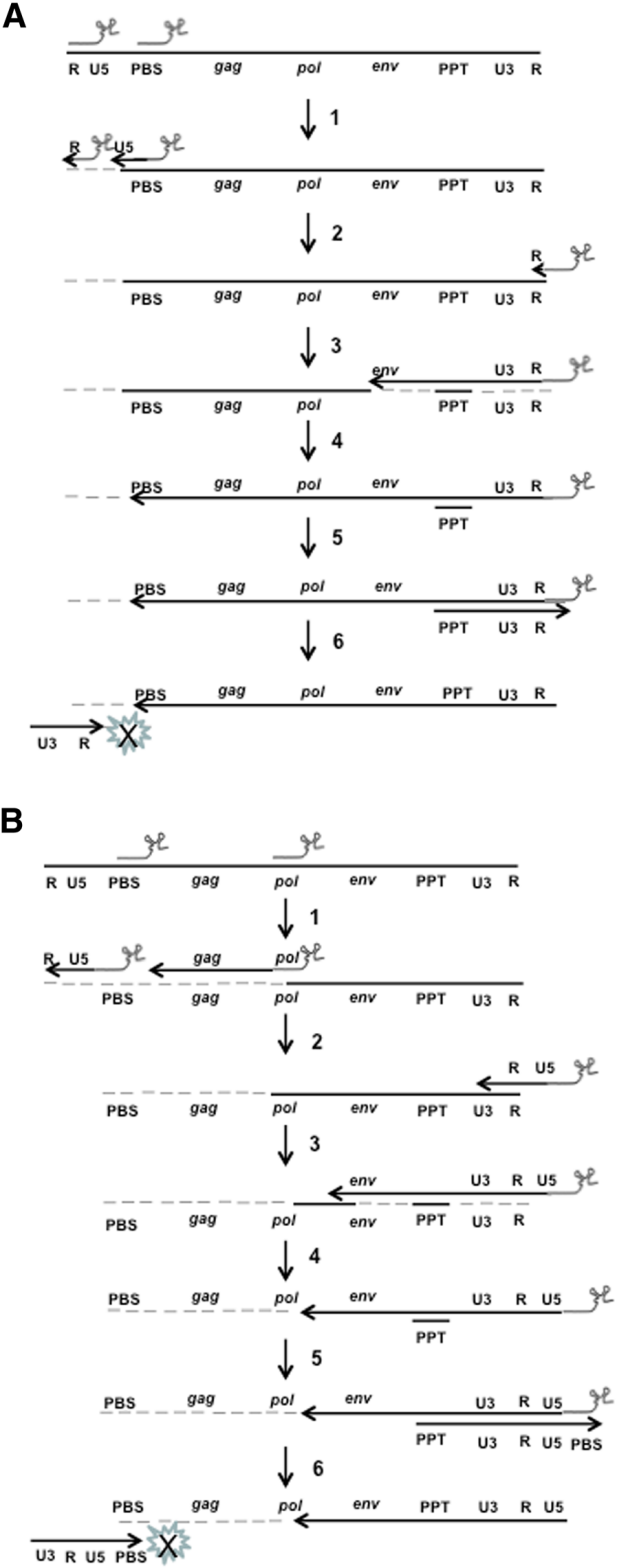
**Proposed mechanisms of disruption of genome conversion of HIV-1.** step 1, tRNA^Lys3^s anneal vRNA and initiate RTions, with vRNA degraded by RNaseH; step 2, strand transfer of (-)ssDNA-tRNA^Lys3^; step 3, (-)strand DNA synthesis proceeds, with vRNA degraded except for PPTs; step 4, (-)strand DNA synthesis proceeds to 5′end of vRNA; step 5, (+)strand DNA synthesis starts with complementary (mutated) PBS copied from tRNA^Lys3^; step 6: tRNA^Lys3^ is removed, and the second strand transfer fail to proceed. **(A)** Mechanisms by mutant tRNA^Lys3^ targeting the TAR. Both tRNA^Lys3^s are encapsidated and initiate RTions, generating two products. One primed by the mutant contain a short 5′ end of the R, but lack sequence from the 3′ end to the PBS (step 1). Following strand transfer, if it happens, synthesis of (-)ssDNA strand proceeds and degrades vRNA (step 3). Simultaneously wild-type tRNA^Lys3^-primed RTion proceeds and degrades vRNA (steps 2–6). Consequently, the integrity of vRNA is disrupted and the second strand transfer cannot proceed (step 6). For the (-)ssDNA primed by the wild-type tRNA^Lys3^, strand transfer may be blocked or proceed at lower efficiency due to lack of 5′ end of the R, and extension of the (-)DNA synthesis cannot proceed due to disrupted integrity of vRNA (step 3). **(B)**. Mechanisms by mutant targeting sites downstream PBS. The mutant targeting the IN-coding region is taken as an example. Similar to **(A)**, both tRNA^Lys3^s initiate RTions with vRNA degraded. The (-)ssDNA primed by mutant lack sequences beyond the PBS (step 1), which disrupts strand transfer. Extension of the (-)stand DNA synthesis by the (-)ssDNA primed by the wild-type tRNA^Lys3^ cannot proceed (step 4) due to disrupted integrity of vRNA (step 1&2), which further disrupts the second strand transfer (step 6).

Upon HIV-1 infection of cells that express mutant tRNA^Lys3^, both wild-type and mutant tRNA^Lys3^ are encapsidated into the progeny virions and are capable of initiating RTions from the PBS and the targeting sites, resulting in aberrant RTion products. Furthermore, due to the RNaseH-mediated degradation of vRNA, a gap between the site being targeted and the PBS is generated. Consequently, integrity of the viral genome is disrupted. This would lead to abortion of the genome conversion and non-productive infection of the cell (Figure 8). Consequently, replication cycle of the virus is bleached.

However, due to competition between the wild-type and mutant tRNA^Lys3^s and presence of high concentrations of the wild-type tRNA^Lys3^ in the cell, some progeny virions may carry the natural set of tRNA^Lys^ without mutant tRNA^Lys3^ where infectivity of this type of progeny virus is not affected. In addition, priming of RTion by the wild-type tRNA^Lys3^ and complete synthesis of the (-)ssDNA before disruption of the viral genome through RTion primed by the mutant tRNA^Lys3^ also may take place in some virions due to encapsidation of insufficient amount of mutant tRNA^Lys3^. This will lead to successful conversion of the viral genome and productive infection of the cell. These possibilities may explain why CEM-SS cells transduced with mutant tRNA^Lys3^ showed significantly reduced support to HIV-1 replication but did not completely eliminate the viral infection. However, impact of these possibilities can be minimized through increasing expression/concentration of the mutant tRNA^Lys3^ in target cells by transduction of multiple copies of the gene, or simultaneous transduction of multiple mutants that target various portions on the HIV-1 genome.

## Conclusions

We demonstrated that the potency of anti-HIV-1 activity of the mutant tRNA^Lys3^ correlated with the length of complementarity between the mutated PBS-binding region and the targeting site, and we showed that increasing the concentration of mutant tRNA^Lys3^ in cells through transduction of multiple copies of the gene further augmented its anti-HIV-1 potency. We also targeted effectively various sites widely distributed in the HIV-1 genome, which would provide an effective means of fighting the evolution of resistance HIV-1 mutants. Because of the specific associations between HIV-1 and tRNA^Lys3^, off-target side effects that are associated with other anti-HIV-1 approaches can be avoided, which may offer significant advantages over conventional anti-HIV-1 methods such as antisense RNA or RNA interference. Inhibition of HIV-1 replication through mutant tRNA^Lys3^ may represent a novel and effective gene therapy approach against HIV-1-associated diseases.

## Methods

### Mutant tRNA construction and cloning

Mutant tRNA^Lys3^s with various lengths of mutations in their PBS-binding region that targeted new sites in HIV-1 genome were generated through PCR-based mutagenesis as previously described [33] with minor modifications. Briefly, a primer including the coding sequence of tRNA^Lys3^ and the desired mutations, as specified in Table 4, was synthesized (integrated DNA techniques, IDT) and used as a template for PCR amplification with primers FatRNA and RatRNA. The PCR product was named fragment A. A second PCR used primers FbtRNA and RbtRNA and human genomic DNA extracted from 293T cells as template, with the PCR product named fragment B. A third fusion-PCR used primers FatRNA and RbtRNA, with fragments A and B as templates, and gave rise to a 234-bp fragment. Using primers FatRNA and RbtRNA, the wild-type tRNA^Lys3^ gene was amplified from human genomic DNA, and a mutant tRNA^Lys3^ gene containing the A58U mutation was amplified from plasmid pPPT-PGK-A58U [31] (a kind gift from Dr. Planelles Vicente, University of Utah). The amplified genes were subsequently cloned into the *SnaB* I restriction site of the plasmid pSV-N2A-GFP [34].

**Table 4:** **Primers used for PCR-based amplification of mutant tRNA**^**Lys3 **^**derivatives**

**Primer name**	**Primer sequence (5′ to 3′)**
FatRNA	CAAGCTTATAAGCAAAAAGTAAAGCTCTCGTGAAG
RatRNA	CGCCATGGGTTTTCGTTTCTGTACG
FbtRNA	GTCTTTGCTTTTGGGTACCGCACT
RbtRNA	GTTCGAAATAATGGGAGGTGGCACGAAACGAC
Mt8TD	GCTCTCGTGAAGA ***TAGACC*** ATAGCTCAGTCGGTAGAGCATCAGACTTTTAATCTGAGGGTC ***G*** A ***GG*** GTTCAAGTCCCT ***C*** T ***GGTCTAA*** GCATGTCTTTGC
Mt10TD	GCTCTCGTGAAGA ***TAGACC*** ATAGCTCAGTCGGTAGAGCATCAGACTTTTAATCTGAGGGTC ***G*** A ***TC*** GTTCAAGTC ***GA*** T ***C*** T ***GGTCTAA*** GCATGTCTTTGC
Mt11TD	GCTCTCGTGAAGA ***TAGACC*** ATAGCTCAGTCGGTAGAGCATCAGACTTTTAATCTGAGGGTC ***G*** A ***TCT*** TTCAAGT ***AGA*** T ***C*** T ***GGTCTAA*** GCATGTCTTTGC
Mt11TD-G44C	GCTCTCGTGAAGA ***TAGACC*** ATAGCTCAGTCGGTAGAGCATCAGACTTTTAATCTGA ***C*** GGTC ***G*** A ***TCT*** TTCAAGT ***AGA*** T ***C*** T ***GGTCTAA*** GCATGTCTTTGC
Mt11TD-A58U	GCTCTCGTGAAGA ***TAGACC*** ATAGCTCAGTCGGTAGAGCATCAGACTTTTAATCTGAGGGTC ***G*** A ***TCT*** TTCA ***T*** GT ***AGA*** T ***C*** T ***GGTCTAA*** GCATGTCTTTGC
Mt13TD	GCTCTCGTGAAGA ***TAGACC*** ATAGCTCAGTCGGTAGAGCATCAGACTTTTAATCTGAGGGTC ***G*** A ***TCT*** TTCAA ***TCAGA*** T ***C*** T ***GGTCTAA*** GCATGTCTTTGC
Int	GCTCTCGTGAAGA ***TTTATT*** ATAGCTCAGTCGGTAGAGCATCAGACTTTTAATCTGAGGGTCCAGGGTTCAAGTCCCTGT ***AATAAAC*** GCATGTCTTTGC
Env	GCTCTCGTGAAGAG ***GTG*** GG ***G*** TAGCTCAGTCGGTAGAGCATCAGACTTTTAATCTGAGGGTC ***AT*** GGGTTCAAGTCCC ***ATT*** TC ***CAC*** C ***C*** GCATGTCTTTGC

Note: mutated bases are shown in bold italic.

To construct the vector with three copies of Mt13TD, the plasmid with one copy was first digested with *Bgl* II and blunted with DNA polymerase I Klenow fragment to insert the second copy, and the resultant plasmid was digested with *Sac* II and blunted to insert the third copy. To construct the vector with six copies, the plasmid with three copies was digested with *Mlu* I and blunted and inserted with a three-copy fragment cut from the same plasmid with *Nhe* I and blunted with Klenow fragment. To construct the vector with nine copies, the plasmid with six copies was digested with *Mlu* I and blunted, and inserted with the three-copy fragment. To construct the vector with twelve copies, the plasmid with nine copies was digested with *Mlu* I and blunted, and inserted with the three-copy fragment. To prevent self-ligation, linearized plasmid DNAs were treated with calf intestinal alkaline phosphatase (New England Biolabs, NEB) as previously described [61] before ligations.

### Delivery of mutant tRNA^Lys3^ into target cells

Retroviral vector were packaged and used to transduce CEM-SS cells as previously described [34,62]. Detection of mutant tRNA^Lys3^ in transduced cells was performed by PCR with primers specific to the mutated regions of the mutants and genomic DNA extracted from the cells as template. Primers (F) 5′-TAGACCATAGCTCAG-3′ and (R) 5′-TGGTTAGACCAGATC-3′ were used for detection of the mutants targeting the TAR; primers (F) 5′-TTTATTATAGCTCAGTC-3′ and (R) 5′-TGGGTTTATTACAGGG-3′ were used for detection of the mutant targeting the *Env* gene; and primers (F) 5′-GGTGGGGTAGCTCAG-3′ and (R) 5′-TGGGGGTGGAGGTGG-3′ were used for detection of the mutant targeting the IN-encoding region. To examine expression of the mutant tRNA^Lys3^s, RT-PCR was used with the same primers. Briefly, total RNA was extracted from transduced CEM-SS cells using the acid guanidinium isothiocyanate/phenol-chloroform method [63]. First strand synthesis was done using MoMuLV RT (NEB) following the vendor’s manual with 100 ng antisense primer specific to the gene to be detected. One microliter from the reaction was used as template for PCR amplification, with the PCR product separated with 2% agarose gel and visualized through ethidium bromide staining.

### Cells and viruses

293T cells and CEM-SS cells were routinely maintained and split as previously described [62]. Replication competent HIV-1 virus was generated through transient transfection of 293T cells with plasmid pHIV-thy (from Dr. Planelles) and used for HIV-1 challenging tests. Primary virus preparation was used to infect CEM-SS cells, and virus-containing supernatant was collected on day 9 when maximal amount of syncytia were observed, aliquoted in 1.0 mL and stored at -80°C until used.

### Cytotoxicity tests

MTT assay [64] was performed as previously described [38] with minor modifications. Briefly, cells non-transduced or transduced with mutant tRNA^Lys3^ were inoculated in triplicates at 1.0 × 10^4^ cells/well in 96-well plate in 100 μl RPMI-1640 medium with 2% FBS including three wells without cell as blanks, and cultured at 37 C with 5% CO_2_. Each well was treated with 10 μl MTT (5 mg/mL) for 4 h at 37 C on day 3 post inoculation, followed by addition of 100 μl DMSO. Plate was gently swirled and left with cover in the dark for 4 hours at room temperature. To measure the absorbance, plate was read at 570 nm using a microplate reader (Beckman Coulter AD340). The optical densities (OD) were compared and used for evaluating cell growth and viability.

A lentiviral vector expressing the firefly luciferase gene and eGFP was constructed through cutting the cDNA of luciferase from pNL-CMV-Luc (from Dr. Planelles) with *Xho* I and *Mlu* I, and ligated into the plasmid pHR-hTNFR-Fc-eGFP [38] that was cut by *Xho* I and *Asc* I. Resultant plasmid, named pHR-luc-eGFP, and pHR-hTNFR-Fc-eGFP were respectively packaged as previously described [65], and used to infect 1.0 × 10^5^ CEM-SS at MOI 10.0. On day 7 following infection, supernatant of the cultured cells were collected and examined for sTNFR-Fc expression as previously described [38]. Test for luciferase activity was done with the dual luciferase assay kit (Promega). Following the vendor’s manual, cell lysates were prepared from 1.0 × 10^6^ cells using 200 μl 1× PLB through the passive lysis method, and 20 μl from each lysate were sampled for the test. Luminescence was measured in a Turner luminometer-96 (Turner Designs, Sunnyvale, CA). The readings, counts per second (CPS), were used to evaluate luciferase activities.

### TCID_50_ assay

TCID_50_ assay was performed as previously described [33] with minor modifications. Briefly, CEM-SS cells at the exponential growth stage were seeded into 96-well plates at 5 × 10^3^ cells/well in 100 μL RPMI1640 medium with 10% heat-inactivated fetal bovine serum (FBS). The HIV-1 virus stock was serially diluted 10-fold with RPMI1640 medium without serum, and 100 μL/well of each virus dilution was inoculated into 4 wells with cells along with control wells receiving the same amount of virus-free medium. The infected cells were examined daily for syncytia formation and TCID_50_ readings were determined on day 15 pi.

### HIV-1 challenge and P24 assay

HIV-1 challenge and P24 assay was done as previously described [58] with minor changes. Briefly, 4 × 10^5^ cells transduced with mutant tRNA^Lys3^ in the exponential growth phase were pelleted with a bench top centrifuge at 3000 rpm for 3 minutes, washed once with 1.0 mL RPMI1640 medium without serum, pelleted, and then resuspended in 1.0 mL of diluted HIV-1 virus at the desired MOI. After adsorption at 37°C for 90 minutes, cells were pelleted and washed for three times with 1.0 ml RPMI1640. After the third washing and pelleting, supernatant was discarded. Cells were resuspended in 6.0 mL RPMI1640 medium containing 10% heat-inactivated FBS, and incubated at 37°C in a T-25 flask. Every 2 or 3 days following the infection, 0.5 mL of cell-free supernatant was collected from the flasks and used for P24 assay through an antigen capture enzyme-linked immunosorbent assay (ELISA) (Coulter Immunology, Hialeah, FL).

### Mutant tRNA^Lys3^ encapsidation assay

To confirm encapsidation of mutant tRNA^Lys3^, retroviral vector plasmid containing the mutants were respectively co-transfected with a three-plasmid HIV-1-based vector system [62]. For negative controls, transfections omitting the packaging plasmid were performed. Supernatant conditioned by transfected cells were collected and titrated on CEM-SS cells. In addition, 35 mL supernatant was concentrated into 0.1 mL through an ultracentrifugation method [34]. Viral RNA was extracted using the QIAamp viral RNA mini kit (Qiagen) with detection of mutant tRNA^Lys3^ from the RNA extractions performed through RT-PCR using the same primers as used for the detection of the mutants form transduced CEM-SS cells.

### Priming assay

To characterize the specificity and efficiency of RTion primed by mutant tRNA^Lys3^, a competitive PCR-based method was employed. Briefly, HIV-1-based vectors with mutant tRNA^Lys3^ were prepared and concentrated as previously described, with 0.1 ml of concentrated vector used to infect 1.0 × 10^6^ CEM-SS cells. For negative controls, infections were done with the same amount of vector that was inactivated by incubation at 65°C for 60 minutes. Infected cells were incubated at 37°C for 12 hours in 4.0 ml RPMI1640 with 10% FBS, and then pelleted and used for DNA extraction as previously described [34]. Twenty nanogram of extracted DNA was used as template for PCR amplification, with primers specified in Table 5. For mutants that targeted the TAR, we used the forward primer named F-tRNA that is specific to the U3 region of HIV-1 3′ LTR (nucleotides 7354–7377 in the transfer plasmid of the HIV-1-based vector system [62]), and a reverse primer specific to the mutated region of each mutant. Where RTion is initiated from the TAR, the PCR products is amplified from the (-) and (+) ssDNAs generated (as illustrated in steps 3–5 in Figure 8), which is 226 bp in size. In case where RTion is initiated from the PBS as previously reported [33], the PCR product is 395 bp in size. For mutants that targeted the IN-encoding region and *Env* gene respectively, two PCR reactions were performed for each mutant. The forward primer, F-tRNA, and a reverse primer specific to the mutated PBS-binding region of the mutant, were used to amplify and detect the (-) and (+) ssDNAs primed from the PBS with the PCR product 395 bp in size. To detect the (-) and (+) ssDNAs primed from their targeting sites, forward primers, named as F-Env and F-Int that were specific to a site 226 bp upstream of their targeting sites, and the same reverse primer, were used. The PCR product is 226 bp in size. Following amplifications, the PCR products were separated and visualized as previously described.

**Table 5:** Primers used for priming assay

**Primer name**	**Primer sequence (5′ to 3′)**
F-tRNA	GGAGGTTTGACAGCCGCCTAGCAT
F-Env	GCAGTAAGTAGTACATGTAATGCAACC
F-Int	TAAAGAATTAAAGAAAATTATAGGACAGGTAAGAG
R-WT-tRNA	GTCCCTGTTCGGGCGCCA
R-Mt8TD	GTCCCTCTGGTCTAACCA
R-Mt10TD	GTCGATCTGGTCTAACCA
R-Mt11TD	GTAGATCTGGTCTAACCA
R-Mt13TD	TCAGATCTGGTCTAACCA
R-env	GTCCCATTTCCACCCCCA
R-int	GTCCCTGTAATAAACCCA

### Real-time PCR and RT-PCR

To examine copy numbers of the Mt13TD gene in cells transduced with the multiple-copy vector constructs, real-time PCR was employed using 20.0 ng genomic DNAs extracted from the cells as template. Primers specific for Mt13TD were (F) 5′-TAGACCATAGCTCAGTCGGTAGAGCATCAG-3′and (R) 5′-TGGTTAGACCAGATCTGATTGAAAGATCG-3′, and primers specific the wild-type tRNA^Lys3^ were (F) 5′-GCCCGGATAGCTCAGTCGGTAGAGCATCAG-3′ and (R) 5′-TGGCGCCCGAACAGGGACTTGAACCCTGG-3′. To examine the relative expression of Mt13TD versus wild-type tRNA^Lys3^, total cellular RNAs were extracted from the cells, with 2.0 μg RNAs used for RTion as previously mentioned, using the wild-type tRNALys3- and Mt13TD-specific reverse primers. Subsequently, copy numbers of the wild-type tRNALys3 and Mt13TD in 1.0 μl of the RTion were respectively determined through real-time PCR.

For real-time PCR analysis, 1.0 μl of genomic DNAs or cDNA were amplified in triplicates in 25 μl reaction volumes with 0.2 μM concentrations of each primer using the IQ SYBR GREEN Super mix (Bio-Rad, Hercules, CA). The principle of the real-time PCR has been described elsewhere [65]. Briefly, following activation of the iTaq™ DNA polymerase for 10 min at 95°C, 40 cycles (15 s at 95°C and 1 min at 72°C) were performed with the iQ5 real-time PCR detection system (Bio-Rad). The positive controls consisted of the retroviral plasmid containing the gene to be tested. As negative control, samples consisting of distilled water were also subjected to the DNA/RNA extraction procedure and the resulting extracts were amplified. Standard graphs of the *CT* values obtained from serial dilutions (10 to 10^6^ copies) of the retroviral plasmids were constructed, and the *CT* values from unknown samples were plotted on the standard curves. Subsequently, copy number of the gene was calculated. Copy numbers of the plasmids and the number of human cells that 20 ng genomic DNAs were extracted from were calculated with the formula as following: number of copies/cells = (amount * 6.022×10^23^)/(length * 1×10^9^ * 650) [http://cels.uri.edu/gsc/cndna.html], with amount referring to the amount of DNA present in ngs and length referring to the length of plasmid or amount of DNA in a single human cell in bp. The length of mutant tRNA^Lys3^-containing retroviral plasmid was 9638 bp and length of DNA from a single human cell is about 6.6x10^9^ bp [http://users.rcn.com/jkimball.ma.ultranet/BiologyPages/G/GenomeSizes.html]. Relative expression of Mt13TD versus wild-type tRNA^Lys3^ was calculated through dividing the copy number of Mt13TD by the copy number of wild-type tRNA^Lys3^.

### Statistical analysis

Origin 6.0 professional software (OriginLab Corporation) was used for two-population t-tests or one-way ANOVA analysis. P < 0.05 was considered statistically significant. * indicates 0.01 < P < 0.05; ** indicates 0.001 < P < 0.01; *** indicates P < 0.001.

## Abbreviations

HIV-1: Human immunodeficiency virus type 1; RTion: Reverse transcription; TAR: Trans-activation response region; PBS: Primer binding site; vRNA: Viral RNA; RT: Reverse transcriptase; R: Repeat region; PPT: Polypurine tracts; LTR: Long terminal repeat; TCID50: Median tissue culture infective dose; MOI: Multiplicity of infection; bp: Base pair; pi: Post infection; LysRS: Lysyl-tRNA synthetase; FBS: Fetal bovine serum; ELISA: Enzyme-linked immunosorbent assay; CPS: Count per second; OD: Optical density.

## Competing interests

The authors declare that they have no competing interests.

## Authors’ contributions

CW participated in designing the study, carried out the experiments, collected and interpreted the data, and wrote the manuscript. VRN participated in coordination of the study and revised the manuscript. YL conceived and designed the study, participated in data analysis and coordination, and revised the manuscript. All authors read and approved the final manuscript.
